# Supplementary material for: Tumour-associated macrophages mediate the invasion and metastasis of bladder cancer cells through CXCL8
Source: PeerJ. 2020 Mar 12;8:e8721. doi: 10.7717/peerj.8721 (PMC7073239; doi:10.7717/peerj.8721)
Supplement: Supplemental Information 2 [file peerj-08-8721-s002.docx]

**Abbreviations**

**TAMs:** Tumour-associated macrophages

**CXCL8:** Chemokine (C-X-C motif) ligand 8

**MMP-9:** matrix metalloproteinase-9

**VEGF:** vascular endothelial growth factor

**PBM:** peripheral blood mononuclear cells

**CM:** conditioned media

**CD163:** hemoglobin scavenger receptor

**CCL2:** (C-C motif) ligand 2

**CSF-1:** colony-stimulating factor 1

**CSF-1R:** Colony stimulating factor-1 receptor

**H&E stained:** hematoxylin and eosin-stained

**BSA:** bovine serum albumin

**DAB:** 3, 3` -diaminobenzidine

**IL-4:** interleukin 4

**ELISA:** Enzyme Linked Immunosorbent Assay

**qRT-PCR:** Quantitative real-time polymerase chain reaction

**EMT:** Epithelial-mesenchymal transition
